# Bedside Endoscopic Ultrasound in Critically Ill patients

**DOI:** 10.1155/2011/529791

**Published:** 2011-06-06

**Authors:** Mehdi Mohamadnejad, Julia K. LeBlanc, Stuart Sherman, Mohammad Al-Haddad, Lee McHenry, Gregory A. Cote, John M. DeWitt

**Affiliations:** ^1^Division of Gastroenterology and Hepatology, School of Medicine, Indiana University, Indianapolis, IN 46202, USA; ^2^Digestive Disease Research Center, Tehran University of Medical Sciences, Tehran 14117, Iran

## Abstract

*Background*. The aim of this study was to evaluate the role and impact of EUS in the management of critically ill patients. *Methods*. We retrospectively identified all patients at our institution over a 68-month period in whom bedside inpatient EUS was performed. EUS was considered to have a significant impact if a new diagnosis was established and/or the findings altered subsequent clinical management. *Results*. Fifteen patients (9 male; mean age 58 ± 15 years) underwent bedside EUS without complications. EUS-FNA (median 4 passes; range 2–7) performed in 12 (80%) demonstrated a malignant mediastinal mass/lymph node (5), pancreatic abscess (1), excluded a pelvic abscess (1), established enlarged gastric folds as benign (1) and excluded malignancy in enlarged mediastinal (1) and porta hepatis adenopathy (1). In two patients, EUS-FNA failed to diagnose mediastinal histoplasmosis (1) and a hemorrhagic pancreatic pseudocyst (1). In three diagnostic exams without FNA, EUS correctly excluded choledocholithaisis (*n* = 1) and cholangiocarcinoma (1), and found gastric varices successfully thrombosed after previous cyanoacrylate injection (1). EUS was considered to have an impact in 13/15 (87%) patients. *Conclusions*. In this series, bedside EUS in critically ill patients was technically feasible, safe and had a major impact on the majority of patients.

## 1. Introduction

Endoscopic ultrasonography (EUS) is an established modality for the diagnosis of esophagogastric, mediastinal, pancreatobiliary, and pelvic lesions. However, it is mainly used for elective procedures in outpatients and occasionally inpatients who are at low risk for complications from moderate or deep sedation. While diagnostic and therapeutic endoscopy procedures such as PEG placement or evaluation of GI bleeding are routinely performed at the bedside in intensive care units (ICUs), the use of EUS for this indication has been described in only two small case series [1, 2]. In this study, we report a single center experience of the indications and impact of bedside EUS on the care of ICU patients.

## 2. Methods

This study was approved by the Institutional Review Board at Clarian Health Partners/Indiana University Hospital. Our prospectively updated endoscopy database was queried to identify all patients who underwent a bedside EUS procedure in an ICU at our hospital between January 2004 and August 2009.

Patients in this cohort were deemed by their treating physicians to require ICU care and also an EUS procedure for evaluation of various pancreaticobiliary, thoracic, or pelvic disorders. To complete these procedures, patients were not deemed stable for transport to our endoscopy unit and therefore required a bedside procedure.

Sedation for these procedures varied depending on the presence or absence of previous endotracheal intubation. If the patient was intubated, intravenous sedatives or narcotics already being administered to the patient were given for the procedure either by intermittent boluses or a temporary increase in the maintenance infusion rate. Alternatively, intermittent boluses of propofol were used. If the patient was not intubated, then sedation was accomplished by intermittent boluses of benzodiazepines and/or opioids. Appropriate cardiorespiratory monitoring was used for all patients. Informed consent was obtained from the patient, patients' relative or power of attorney in all cases.

All procedures were performed by or under the supervision of one of five experienced attending endosonographers. If a radial echoendoscope was required, examination was initiated with an Olympus GF-UE160-AL5 radial echoendoscope (Olympus America, Inc., Center Valley, PA, USA). Curvilinear array endosonography was performed using an Olympus GF-UC30P or Olympus GF-UC140P-AL5 (Olympus America, Inc., Center Valley, PA, USA) echoendoscope. EUS-FNA was performed at the discretion of the endosonographer and if performed was obtained using a 19 or a 22-gauge EUSN-1, EUSN-2, EUSN-3, or Echotip Ultra needle (Cook Medical Inc., Winston-Salem, NC, USA) or EZ-Shot needle (Olympus America, Inc., Center Valley, PA, USA). Doppler examination was used to ensure the absence of intervening vascular structures along the anticipated needle path. Depending on the amount of blood anticipated during tissue sampling, full, partial, or no suction was applied. When a cytopathologist and portable microscope were available on site for preliminary diagnostic interpretations and assessment of specimen adequacy, FNA samples were expressed onto a glass slide and two smear preparations were made. One slide preparation was air-dried and stained with a modified Giemsa stain for on-site review, while the other slide was alcohol-fixed and stained by the Papanicolaou method. In the absence of on-site pathology review, samples were only alcohol-fixed for future staining and interpretation. EUS-FNA was repeated until a definitive diagnosis was made or the endosonographers felt that further sampling would not likely increase yield. Additional FNA passes were made when either (1) a cellblock preparation was potentially required for immunostains or (2) microbiology stains and cultures were required for potential sites of infections. Definitive cytopathologic diagnoses were given only after complete staining and subsequent final interpretation was provided. Hospital records and endoscopy charts were reviewed after the procedure to assess for any short-term complications and clinical followup.

For study purposes, EUS was considered to have a significant impact if a new diagnosis was established and/or the findings altered subsequent management. EUS was not considered to have an impact if a diagnosis was incorrectly interpreted or missed. When available, the final clinical diagnosis for each patient was made by autopsy or surgery. In the absence of either, the results of EUS-FNA and clinical followup were used.

## 3. Results

### 3.1. Study Population

Fifteen patients (9 male; mean age: 58 ± 15 years) were included. Twelve were from American Society of Anesthesiology (ASA) class III and three were from ASA class IV. Eight (53%) were under mechanical ventilation during EUS. Bed location for these 15 patients included Medical ICU in nine, Surgical ICU in three, Organ Transplant ICU in two, and Neurosurgical ICU in one. Reasons for ICU admission were respiratory failure in seven, severe sepsis in three, severe pancreatitis in two, cardiac arrest in one, liver failure in one, and gastrointestinal bleeding in one. Past history of severe underlying disorder in seven patients included alcoholic cirrhosis in two, retroperitoneal fibrosis in one, renal and pancreas transplant in one, renal transplant in one, Hodgkin's disease in one, and bladder cancer in one.

### 3.2. EUS Findings

Indications for EUS were mediastinal mass or adenopathy (*n* = 7), suspected pancreas mass or abscess (*n* = 3), evaluation of suspected choledocholithiasis or biliary obstruction (*n* = 2), suspected gastric mass (*n* = 1), gastric variceal bleeding (*n* = 1), and suspected pelvic abscess (*n* = 1).

Bedside EUS was technically successful and completed in all patients without complications. Twelve (80%) patients underwent EUS-FNA (median 4 passes; range 2–7) and a cytopathologist was available on site for preliminary interpretations in 8 of 12 (66.7%). The findings and results of the EUS exams are summarized in Table 1.

EUS-FNA provided an initial diagnosis of a malignant mediastinal mass/lymph node in five including nonsmall cell lung cancer (NSCLC) in two, small cell lung cancer (SCLC) in one, metastatic adenocarcinoma in one, and non-Hodgkin's lymphoma (NHL) in one. Additionally, EUS-FNA diagnosed a pancreas abscess (*n* = 1), excluded a pelvic abscess (*n* = 1), and established enlarged gastric folds (*n* = 1) and lymph nodes in the mediastinum (*n* = 1) and portahepatis region (*n* = 1) as benign. In the three diagnostic exams without FNA, EUS excluded both choledocholithaisis (*n* = 1) and a extrahepatic bile duct tumor (*n* = 1), and found gastric varices were successfully thrombosed after previous cyanoacrylate injection (*n* = 1).

EUS failed to establish a correct diagnosis in two patients. In one (patient 2, Table 1), EUS-FNA of a heterogeneous retroperitoneal mass was reported as suspicious for lymphoma, but autopsy showed the lesion was a hemorrhagic pancreatic pseudocyst in the setting of systemic amyloidosis. In the second case (patient 14, Table 1), EUS-FNA of a mediastinal mass incorrectly revealed necrosis but was later confirmed after surgery as histoplasmosis. EUS was therefore considered to have an impact in 13 of 15 (87%) patients, including 10 of 12 (83%) who had EUS-FNA and all the three (100%) who underwent diagnostic EUS alone.

Six (40%) patients died during their ICU admission and the remaining nine were discharged. Five of these died within three months of hospitalization while four remained alive a median 321 days (range: 209–706 days) after discharge. No short-term complications were noted during or immediately after EUS.

## 4. Discussion

There are limited data previously published in two small series that describe the utility of bedside EUS in critically ill patients. Fritscher-Ravens et al. [1] reported the bedside use of EUSFNA in three patients to diagnose a mediastinal abscess following tracheotomy (*n* = 1), relieve pressure from a paratracheal hematoma compressing the right main bronchus (*n* = 1) and diagnose lung cancer in a patient awaiting cardiac transplantation (*n* = 1). The authors concluded that bedside EUS-FNA was feasible and could offer an alternative in life-threatening situations.

Varadarajulu et al. [2] recently described the use and positive clinical impact of EUS in six patients to establish a diagnosis of choledocholithiasis (*n* = 1), mediastinal abscess (*n* = 1), and pancreatic abscess (*n* = 1) in 3 patients and its use in two other patients to rule out the presence of choledocholithiasis (*n* = 1) and a pancreatic pseudocyst (*n* = 1). The authors also reported the successful use of beside EUS-guided transmural drainage of a pancreatic pseudocyst and mediastinal abscess.

In the current series, we found that bedside EUS had a clinical impact in 13 of 15 critically ill patients evaluated. A new diagnosis was established in 6 cases and precluded the need for further studies or intervention in seven others. We found that the most common clinical impact made by EUS was providing a tissue diagnosis of malignancy. However, as the other two reported series [1, 2] found, diagnostic EUS provided important clinical information as well. One of our patients underwent transrectal EUS during which sampling of a pelvic fluid collection correctly excluded a suspected abscess. To our knowledge, this case represents the first description of bedside rectal EUS.

The current study represents the largest series to date detailing the use of bedside EUS and confirms its utility and impact in this patient population. Nevertheless our study is limited by its retrospective design and limited followup in some patients. Further prospective studies involving larger numbers of patients are needed to further define the role of EUS in critically ill patients.

In conclusion, bedside EUS in ICU patients is safe and feasible. In the current series, it had a significant impact on the management of the majority of patients evaluated. Therefore, endosonographers should consider using EUS in ICU patients when clinically indicated. Given the expanding field of EUS, we postulate that therapeutic procedures such as pancreatic necrosectomy [3–5] and cholecystoenterostomy [6] could be performed at the bedside in these patients as an alternative to surgical or interventional radiology procedures. 

## Figures and Tables

**Table 1:** EUS findings, final diagnoses, and outcomes of the patients.

Patient	Age/gender	Indication for EUS	EUS finding	Number of passes (FNA)	On-site cytologist available	Cytological finding	Final diagnosis	Impact of EUS on management	Patient's outcome
1	74/F	Suspected pancreatic mass on CT	Solid-cystic mass at BOP	3	Yes	Acute inflammation^a^	Necrotizing pancreatitis & pancreatic abscess	Yes	Died*
2	66/M	Suspected pancreatic mass on CT	Heterogenous mass in retroperitoneum	4	Yes	Suspicious for lymphoma	Systemic Amyloidosis & hemorrhagic pancreatic pseudocyst	No	Died^†^
3	50/M	Suspected CBD stone	No CBD stone	NA	N/A	NA	UTI & Sepsis	Yes	Alive
4	50/F	Suspected gastric mass	Focal thickening of gastric mucosa (no tumor)	3	No	Benign epithelium	Severe Pancreatitis^b^	Yes	Died^†^
5	72/M	Suspected extrahepatic cholestasis	No CBD tumor or stone	NA	N/A	NA	Liver failure^c^	Yes	Died*
6	76/M	Mediastinal LN on CT	Subcarinal LN	5	No	Reactive LN	Severe pneumonia	Yes	Died*
7	54/M	Evaluating gastric varices	Thrombosed gastric varices	NA	N/A	NA	Gastric variceal bleeding^d^	Yes	Alive
8	52/M	Suspected pelvic abscess	Post-surgical cyst	2	No	Hypocellular sample^e^	Hemorrhagic pancreatitis & sepsis	Yes	Died^†^
9	61/F	Enlarging pancreatic mass	Large portahepatis LN^f^	3	No	Reactive LN	Respiratory failure due to pneumonia	Yes^g^	Alive
10	37/F	Mediastinal mass on CT	Mediastinal mass	3	Yes	Nonsmall cell carcinoma	NSCLC with mediastinal involvement	Yes	Died*
11	43/M	Mediastinal adenopathy on CT	Paraesophageal LN	6	Yes	Adenocarcinoma	Metastatic adenocarcinoma	Yes	Died*
12	58/M	Mediastinal mass on CT	Mediastinal mass	4	Yes	SCC	NSCLC	Yes	Died^†^
13	76/M	Mediastinal adenopathy on CT	Pleural effusion; celiac & perigastric LN	7	Yes	Non-Hodgkin's lymphoma	Non-Hodgkin's lymphoma	Yes	Died^†^
14	28/F	Mediastinal adenopathy on CT	Mediastinal mass	5	Yes	Necrosis	Histoplasmosis	No	Alive
15	67/F	Mediastinal mass on CT	LN in aortopulmonary window	4	Yes	Small cell carcinoma	Small cell lung cancer	Yes	Died^†^

Abbreviations: BOP: body of pancreas; CBD: common bile duct; NA: not applicable; UTI: urinary tract infection; LN: lymph node; NSCLC: nonsmall cell carcinoma; SCC: squamous cell carcinoma.

*Died a few months after discharge from ICU.

^†^Died while was in ICU.

^a^Enterococcus grew on culture of fine needle aspirate demonstrating pancreas abscess.

^b^Patient's final diagnosis was severe pancreatitis of allograft pancreas, superior mesenteric vein and superior mesenteric artery thromboses, and abdominal compartment syndrome.

^c^Liver failure due to alcoholic cirrhosis.

^d^Patient had gastric variceal bleeding due to alcoholic cirrhosis. He underwent EUS for possible EUS-guided injection of cyanoacrylate, but the varices were found thrombosed from the cyanoacrylate injection one week before.

^e^Culture of fine needle aspirate was negative. Autopsy showed no pelvic abscess.

^f^Very large portahepatis LN with heterogeneous echotexture suggestive of recent bleeding.

^g^EUS-FNA had significant impact on the patient management through excluding malignancy.
